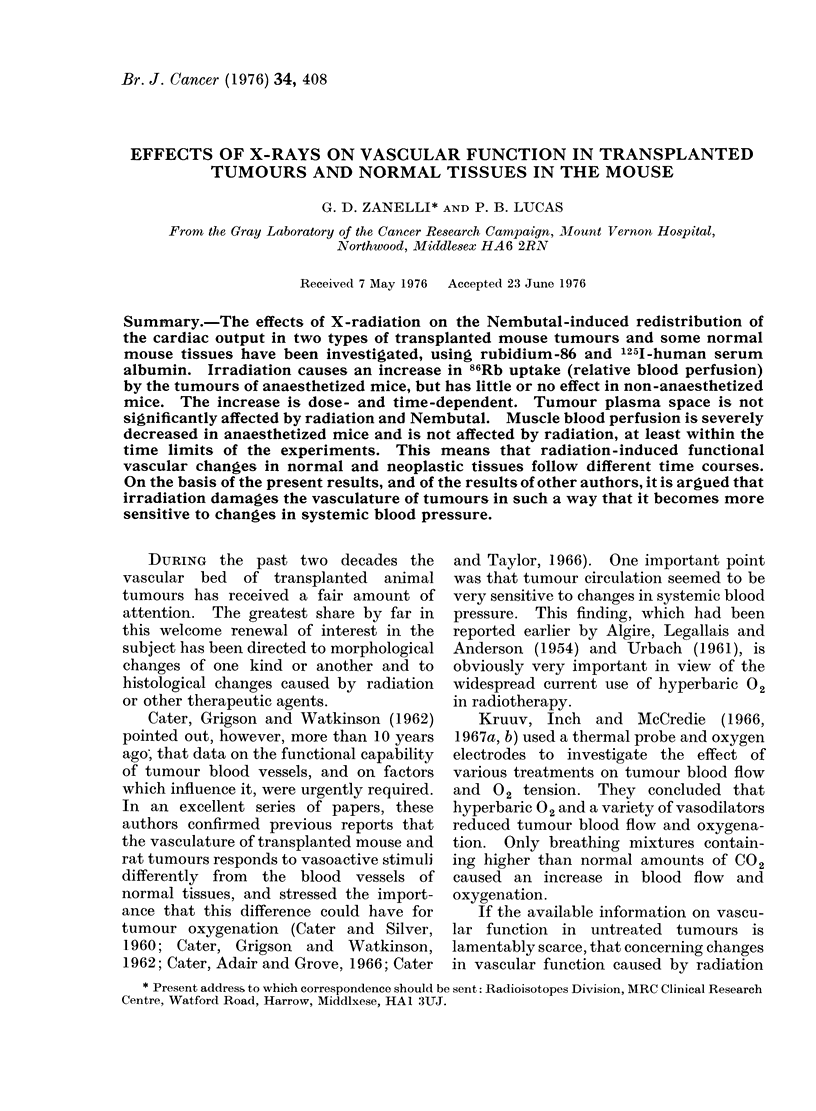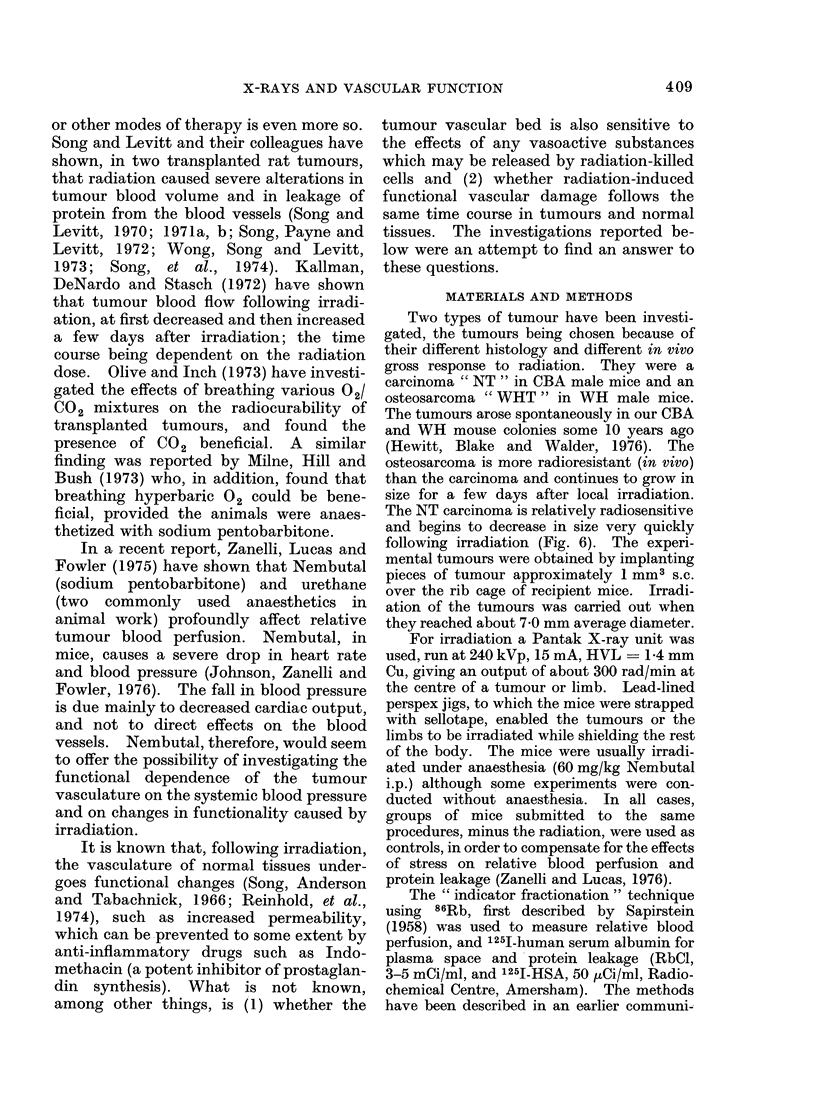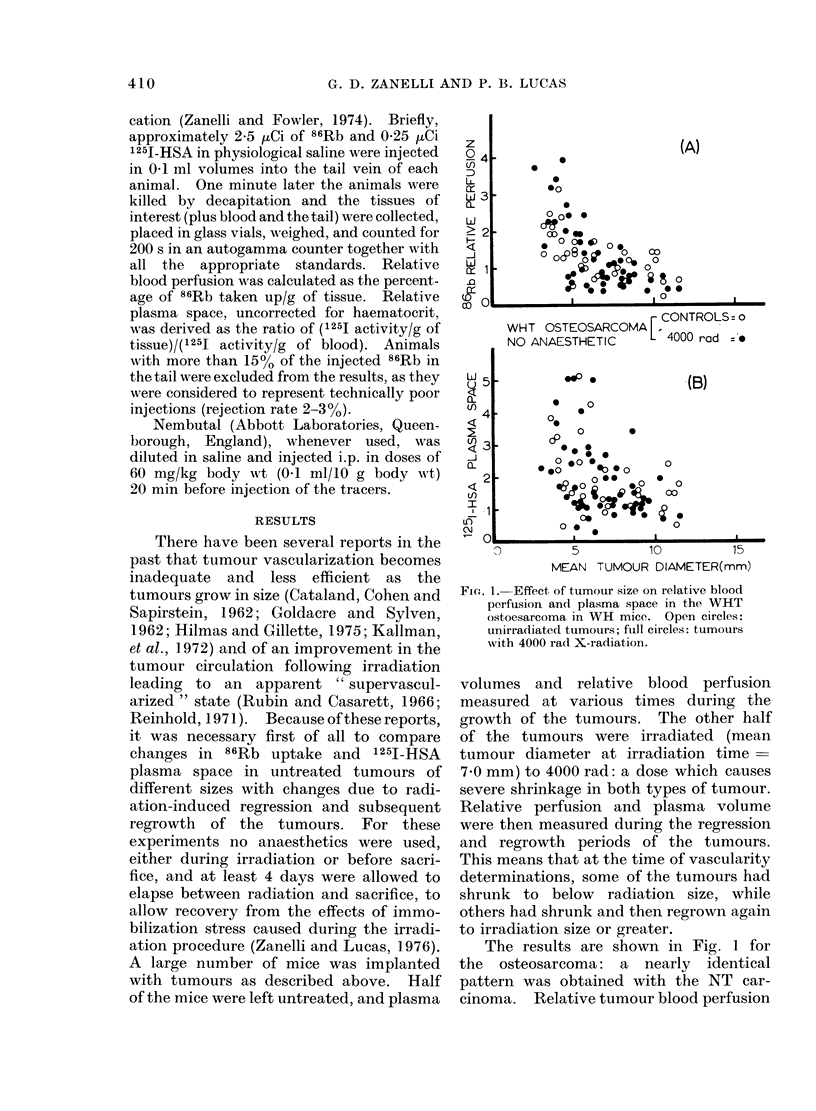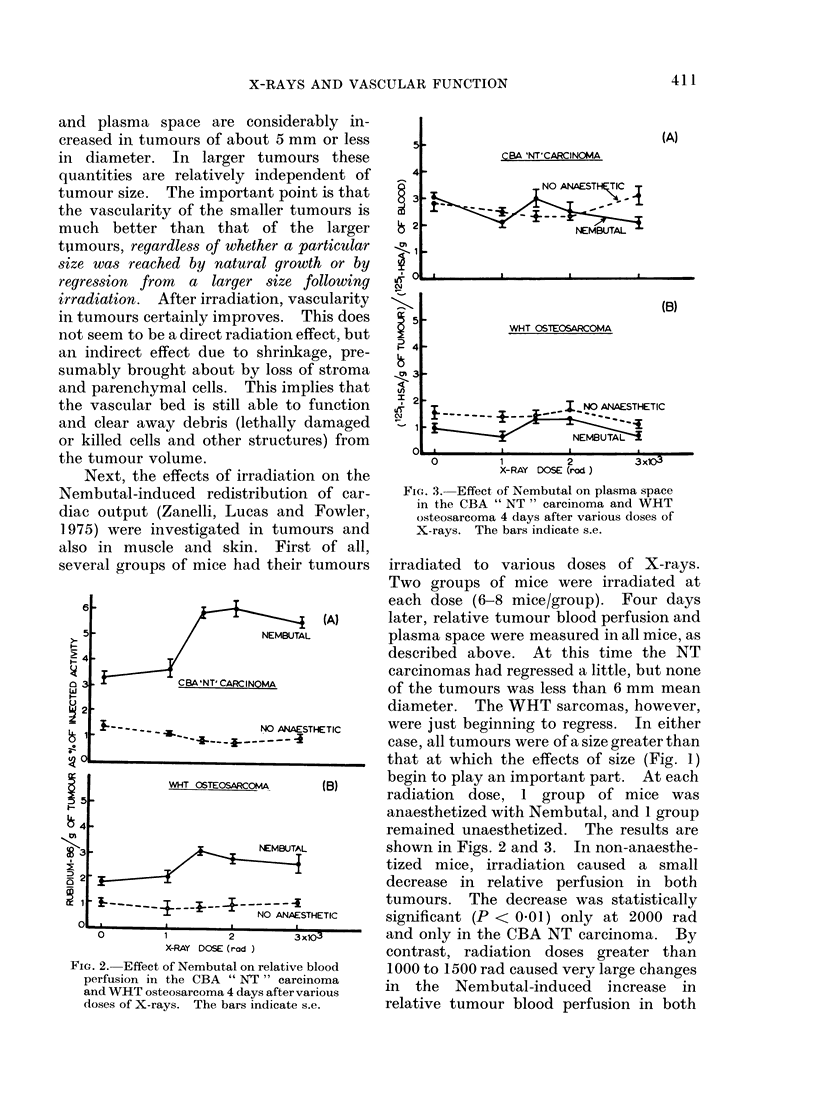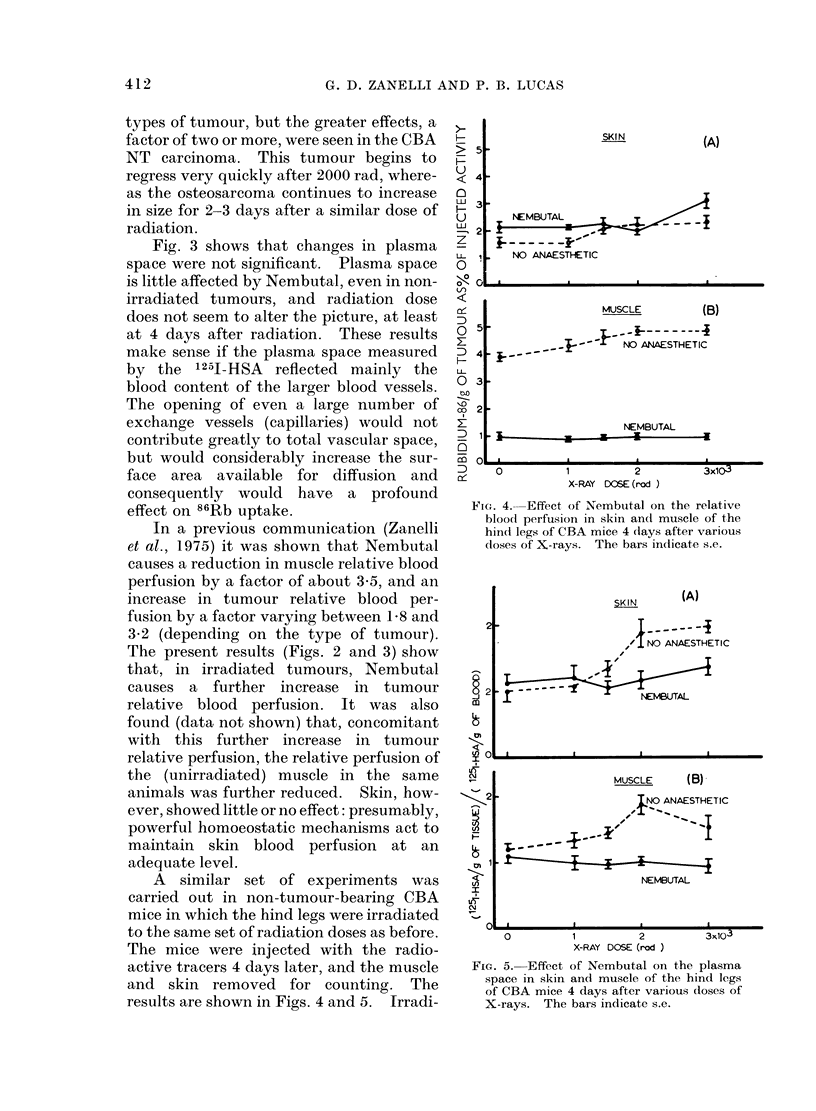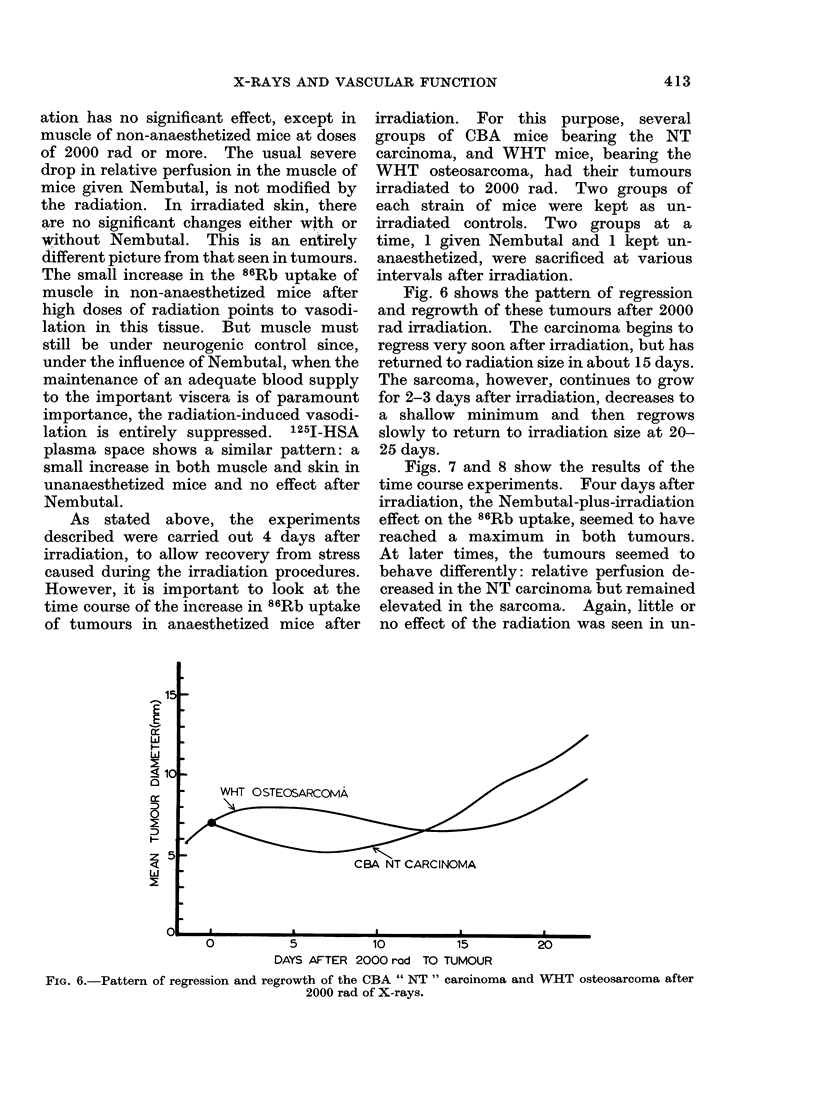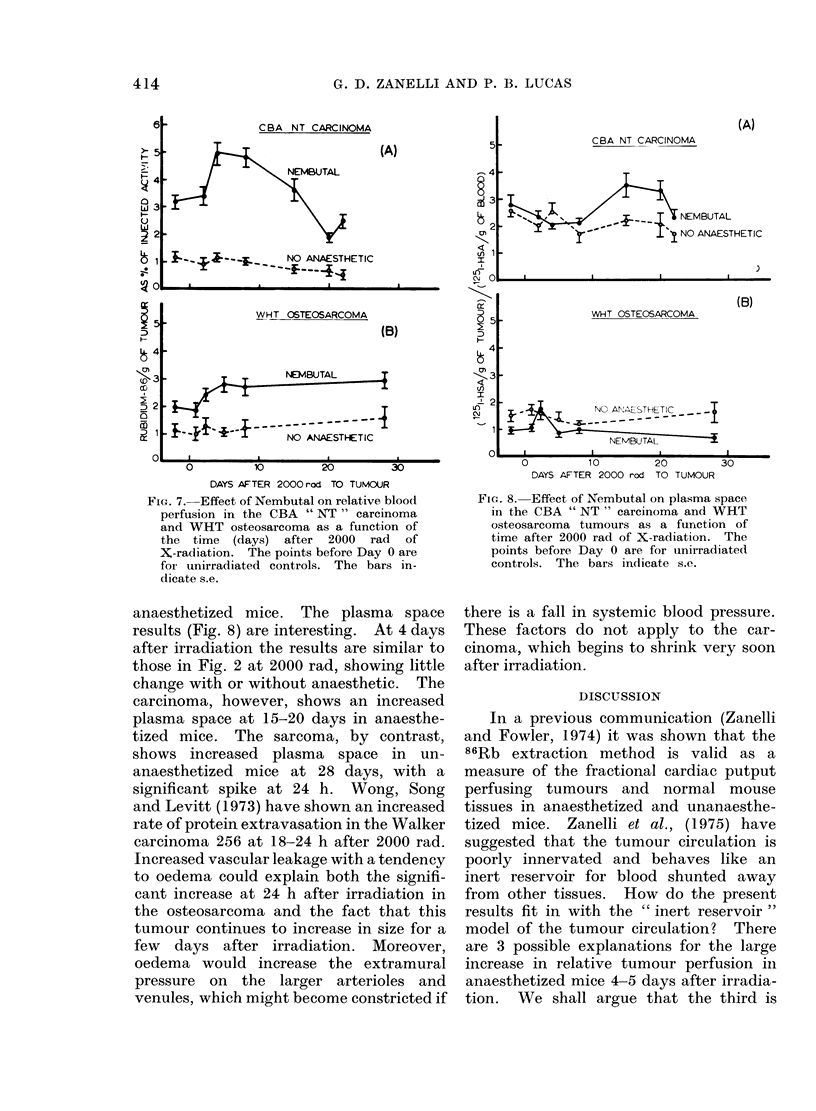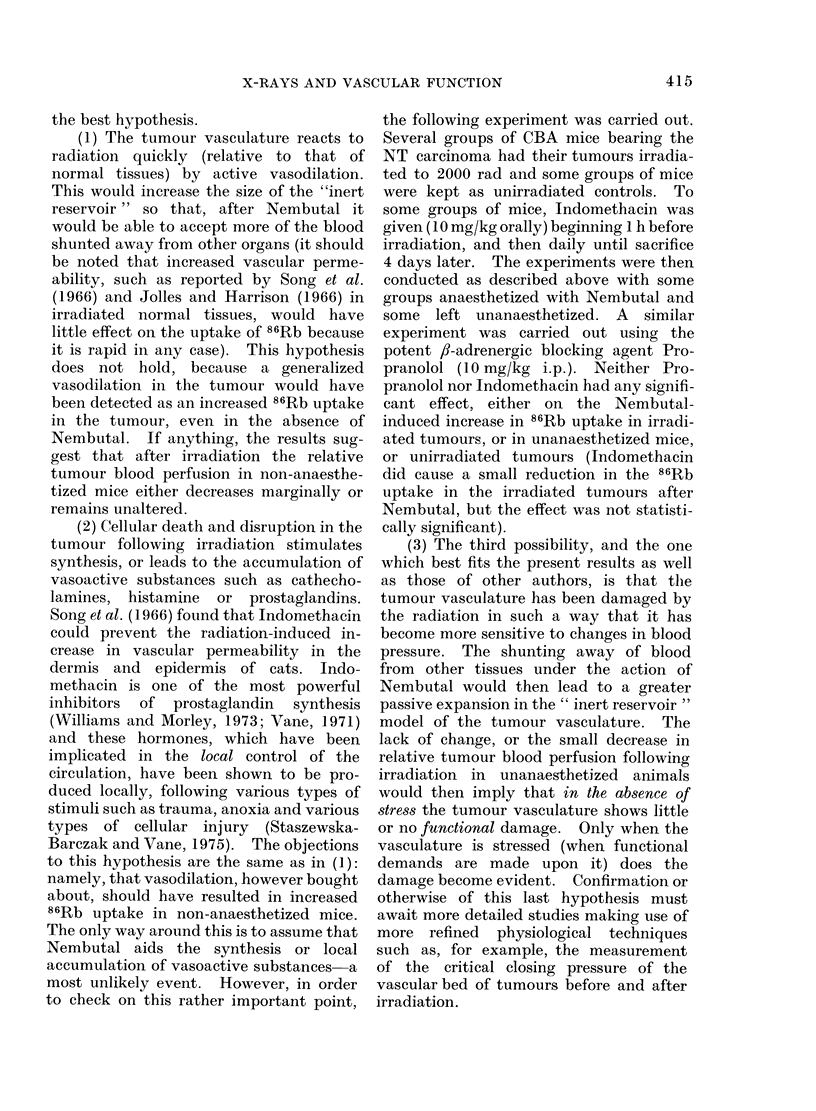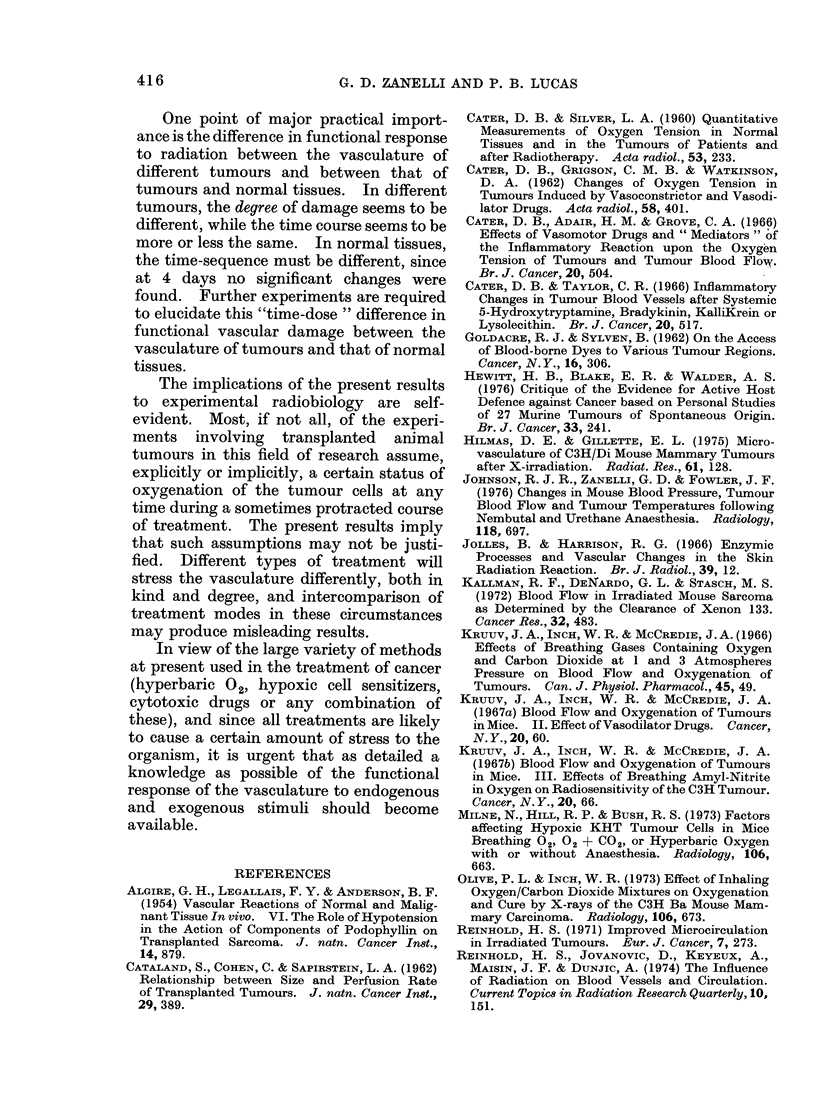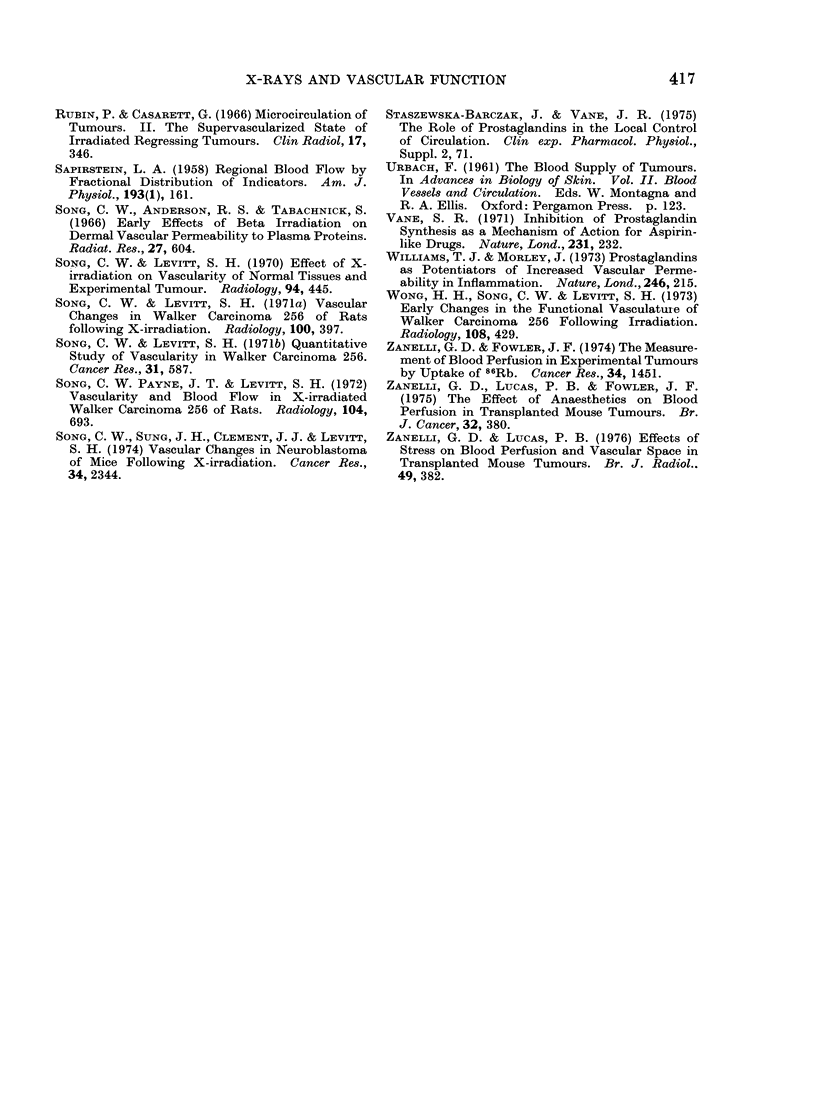# Effects of X-rays on vascular function in transplanted tumours and normal tissues in the mouse.

**DOI:** 10.1038/bjc.1976.186

**Published:** 1976-10

**Authors:** G. D. Zanelli, P. B. Lucas

## Abstract

The effects of X-radiation on the Nembutal-induced redistribution of the cardiac output in two types of transplanted mouse tumours and some normal mouse tissues have been investigated, using rubidium-86 and 125I-human serum albumin. Irradiation causes an increase in 86Rb uptake (relative blood perfusion) by the tumours of anaesthetized mice, but has little or no effect in non-anaesthetized mice. The increase is dose- and time-dependent. Tumour plasma space is not significantly affected by radiation and Nembutal. Muscle blood perfusion is severely decreased in anaesthetized mice and is not affected by radiation, at least within the time limits of the experiments. This means that radiation-induced functional vascular changes in normal and neoplastic tissues follow different time courses. On the basis of the present results, and of the results of other authors, it is argued that irradiation damages the vasculature of tumours in such a way that it becomes more sensitive to changes in systemic blood pressure.


*Br. J. Cancer* (1976) **34**, 408

### EFFECTS OF X-RAYS ON VASCULAR FUNCTION IN TRANSPLANTED TUMOURS AND NORMAL TISSUES IN THE MOUSE

G. D. ZANELLI* AND P. B. LUCAS

*From the Gray Laboratory of the Cancer Research Campaign, Mount Vernon Hospital, Northwood, Middlesex HA6 2RN*

Received 7 May 1976 Accepted 23 June 1976

**Summary.—The effects of X-radiation on the Nembutal-induced redistribution of the cardiac output in two types of transplanted mouse tumours and some normal mouse tissues have been investigated, using rubidium-86 and $^{125}$I-human serum albumin. Irradiation causes an increase in $^{86}$Rb uptake (relative blood perfusion) by the tumours of anaesthetized mice, but has little or no effect in non-anaesthetized mice. The increase is dose- and time-dependent. Tumour plasma space is not significantly affected by radiation and Nembutal. Muscle blood perfusion is severely decreased in anaesthetized mice and is not affected by radiation, at least within the time limits of the experiments. This means that radiation-induced functional vascular changes in normal and neoplastic tissues follow different time courses. On the basis of the present results, and of the results of other authors, it is argued that irradiation damages the vasculature of tumours in such a way that it becomes more sensitive to changes in systemic blood pressure.**

DURING the past two decades the vascular bed of transplanted animal tumours has received a fair amount of attention. The greatest share by far in this welcome renewal of interest in the subject has been directed to morphological changes of one kind or another and to histological changes caused by radiation or other therapeutic agents.

Cater, Grigson and Watkinson (1962) pointed out, however, more than 10 years ago, that data on the functional capability of tumour blood vessels, and on factors which influence it, were urgently required. In an excellent series of papers, these authors confirmed previous reports that the vasculature of transplanted mouse and rat tumours responds to vasoactive stimuli differently from the blood vessels of normal tissues, and stressed the importance that this difference could have for tumour oxygenation (Cater and Silver, 1960; Cater, Grigson and Watkinson, 1962; Cater, Adair and Grove, 1966; Cater and Taylor, 1966). One important point was that tumour circulation seemed to be very sensitive to changes in systemic blood pressure. This finding, which had been reported earlier by Algire, Legallais and Anderson (1954) and Urbach (1961), is obviously very important in view of the widespread current use of hyperbaric $O_2$ in radiotherapy.

Kruuv, Inch and McCredie (1966, 1967*a*, *b*) used a thermal probe and oxygen electrodes to investigate the effect of various treatments on tumour blood flow and $O_2$ tension. They concluded that hyperbaric $O_2$ and a variety of vasodilators reduced tumour blood flow and oxygenation. Only breathing mixtures containing higher than normal amounts of $CO_2$ caused an increase in blood flow and oxygenation.

If the available information on vascular function in untreated tumours is lamentably scarce, that concerning changes in vascular function caused by radiation

* Present address to which correspondence should be sent: Radioisotopes Division, MRC Clinical Research Centre, Watford Road, Harrow, Middlxese, HA1 3UJ.

or other modes of therapy is even more so. Song and Levitt and their colleagues have shown, in two transplanted rat tumours, that radiation caused severe alterations in tumour blood volume and in leakage of protein from the blood vessels (Song and Levitt, 1970; 1971a, b; Song, Payne and Levitt, 1972; Wong, Song and Levitt, 1973; Song, *et al.*, 1974). Kallman, DeNardo and Stasch (1972) have shown that tumour blood flow following irradiation, at first decreased and then increased a few days after irradiation; the time course being dependent on the radiation dose. Olive and Inch (1973) have investigated the effects of breathing various $O_2$/$CO_2$ mixtures on the radiocurability of transplanted tumours, and found the presence of $CO_2$ beneficial. A similar finding was reported by Milne, Hill and Bush (1973) who, in addition, found that breathing hyperbaric $O_2$ could be beneficial, provided the animals were anaesthetized with sodium pentobarbitone.

In a recent report, Zanelli, Lucas and Fowler (1975) have shown that Nembutal (sodium pentobarbitone) and urethane (two commonly used anaesthetics in animal work) profoundly affect relative tumour blood perfusion. Nembutal, in mice, causes a severe drop in heart rate and blood pressure (Johnson, Zanelli and Fowler, 1976). The fall in blood pressure is due mainly to decreased cardiac output, and not to direct effects on the blood vessels. Nembutal, therefore, would seem to offer the possibility of investigating the functional dependence of the tumour vasculature on the systemic blood pressure and on changes in functionality caused by irradiation.

It is known that, following irradiation, the vasculature of normal tissues undergoes functional changes (Song, Anderson and Tabachnick, 1966; Reinhold, *et al.*, 1974), such as increased permeability, which can be prevented to some extent by anti-inflammatory drugs such as Indomethacin (a potent inhibitor of prostaglandin synthesis). What is not known, among other things, is (1) whether the tumour vascular bed is also sensitive to the effects of any vasoactive substances which may be released by radiation-killed cells and (2) whether radiation-induced functional vascular damage follows the same time course in tumours and normal tissues. The investigations reported below were an attempt to find an answer to these questions.

### MATERIALS AND METHODS

Two types of tumour have been investigated, the tumours being chosen because of their different histology and different *in vivo* gross response to radiation. They were a carcinoma " NT " in CBA male mice and an osteosarcoma " WHT " in WH male mice. The tumours arose spontaneously in our CBA and WH mouse colonies some 10 years ago (Hewitt, Blake and Walder, 1976). The osteosarcoma is more radioresistant (*in vivo*) than the carcinoma and continues to grow in size for a few days after local irradiation. The NT carcinoma is relatively radiosensitive and begins to decrease in size very quickly following irradiation (Fig. 6). The experimental tumours were obtained by implanting pieces of tumour approximately 1 $mm^3$ s.c. over the rib cage of recipient mice. Irradiation of the tumours was carried out when they reached about 7·0 mm average diameter.

For irradiation a Pantak X-ray unit was used, run at 240 kVp, 15 mA, HVL = 1·4 mm Cu, giving an output of about 300 rad/min at the centre of a tumour or limb. Lead-lined perspex jigs, to which the mice were strapped with sellotape, enabled the tumours or the limbs to be irradiated while shielding the rest of the body. The mice were usually irradiated under anaesthesia (60 mg/kg Nembutal i.p.) although some experiments were conducted without anaesthesia. In all cases, groups of mice submitted to the same procedures, minus the radiation, were used as controls, in order to compensate for the effects of stress on relative blood perfusion and protein leakage (Zanelli and Lucas, 1976).

The " indicator fractionation " technique using $^{86}$Rb, first described by Sapirstein (1958) was used to measure relative blood perfusion, and $^{125}$I-human serum albumin for plasma space and protein leakage (RbCl, 3–5 mCi/ml, and $^{125}$I-HSA, 50 $\mu$Ci/ml, Radiochemical Centre, Amersham). The methods have been described in an earlier communication (Zanelli and Fowler, 1974). Briefly, approximately 2·5 $\mu$Ci of $^{86}$Rb and 0·25 $\mu$Ci $^{125}$I-HSA in physiological saline were injected in 0·1 ml volumes into the tail vein of each animal. One minute later the animals were killed by decapitation and the tissues of interest (plus blood and the tail) were collected, placed in glass vials, weighed, and counted for 200 s in an autogamma counter together with all the appropriate standards. Relative blood perfusion was calculated as the percentage of $^{86}$Rb taken up/g of tissue. Relative plasma space, uncorrected for haematocrit, was derived as the ratio of ($^{125}$I activity/g of tissue)/($^{125}$I activity/g of blood). Animals with more than 15% of the injected $^{86}$Rb in the tail were excluded from the results, as they were considered to represent technically poor injections (rejection rate 2–3%).

Nembutal (Abbott Laboratories, Queenborough, England), whenever used, was diluted in saline and injected i.p. in doses of 60 mg/kg body wt (0·1 ml/10 g body wt) 20 min before injection of the tracers.

### RESULTS

There have been several reports in the past that tumour vascularization becomes inadequate and less efficient as the tumours grow in size (Cataland, Cohen and Sapirstein, 1962; Goldacre and Sylven, 1962; Hilmas and Gillette, 1975; Kallman, *et al.*, 1972) and of an improvement in the tumour circulation following irradiation leading to an apparent "supervascularized" state (Rubin and Casarett, 1966; Reinhold, 1971). Because of these reports, it was necessary first of all to compare changes in $^{86}$Rb uptake and $^{125}$I-HSA plasma space in untreated tumours of different sizes with changes due to radiation-induced regression and subsequent regrowth of the tumours. For these experiments no anaesthetics were used, either during irradiation or before sacrifice, and at least 4 days were allowed to elapse between radiation and sacrifice, to allow recovery from the effects of immobilization stress caused during the irradiation procedure (Zanelli and Lucas, 1976). A large number of mice was implanted with tumours as described above. Half of the mice were left untreated, and plasma

FIG. 1.—Effect of tumour size on relative blood perfusion and plasma space in the WHT ostoesarcoma in WH mice. Open circles: unirradiated tumours; full circles: tumours with 4000 rad X-radiation.

volumes and relative blood perfusion measured at various times during the growth of the tumours. The other half of the tumours were irradiated (mean tumour diameter at irradiation time = 7·0 mm) to 4000 rad: a dose which causes severe shrinkage in both types of tumour. Relative perfusion and plasma volume were then measured during the regression and regrowth periods of the tumours. This means that at the time of vascularity determinations, some of the tumours had shrunk to below radiation size, while others had shrunk and then regrown again to irradiation size or greater.

The results are shown in Fig. 1 for the osteosarcoma: a nearly identical pattern was obtained with the NT carcinoma. Relative tumour blood perfusion and plasma space are considerably increased in tumours of about 5 mm or less in diameter. In larger tumours these quantities are relatively independent of tumour size. The important point is that the vascularity of the smaller tumours is much better than that of the larger tumours, *regardless of whether a particular size was reached by natural growth or by regression from a larger size following irradiation.* After irradiation, vascularity in tumours certainly improves. This does not seem to be a direct radiation effect, but an indirect effect due to shrinkage, presumably brought about by loss of stroma and parenchymal cells. This implies that the vascular bed is still able to function and clear away debris (lethally damaged or killed cells and other structures) from the tumour volume.

Next, the effects of irradiation on the Nembutal-induced redistribution of cardiac output (Zanelli, Lucas and Fowler, 1975) were investigated in tumours and also in muscle and skin. First of all, several groups of mice had their tumours

FIG. 2.—Effect of Nembutal on relative blood perfusion in the CBA " NT " carcinoma and WHT osteosarcoma 4 days after various doses of X-rays. The bars indicate s.e.

FIG. 3.—Effect of Nembutal on plasma space in the CBA " NT " carcinoma and WHT osteosarcoma 4 days after various doses of X-rays. The bars indicate s.e.

irradiated to various doses of X-rays. Two groups of mice were irradiated at each dose (6–8 mice/group). Four days later, relative tumour blood perfusion and plasma space were measured in all mice, as described above. At this time the NT carcinomas had regressed a little, but none of the tumours was less than 6 mm mean diameter. The WHT sarcomas, however, were just beginning to regress. In either case, all tumours were of a size greater than that at which the effects of size (Fig. 1) begin to play an important part. At each radiation dose, 1 group of mice was anaesthetized with Nembutal, and 1 group remained unaesthetized. The results are shown in Figs. 2 and 3. In non-anaesthetized mice, irradiation caused a small decrease in relative perfusion in both tumours. The decrease was statistically significant ($P < 0{\cdot}01$) only at 2000 rad and only in the CBA NT carcinoma. By contrast, radiation doses greater than 1000 to 1500 rad caused very large changes in the Nembutal-induced increase in relative tumour blood perfusion in both types of tumour, but the greater effects, a factor of two or more, were seen in the CBA NT carcinoma. This tumour begins to regress very quickly after 2000 rad, whereas the osteosarcoma continues to increase in size for 2–3 days after a similar dose of radiation.

Fig. 3 shows that changes in plasma space were not significant. Plasma space is little affected by Nembutal, even in non-irradiated tumours, and radiation dose does not seem to alter the picture, at least at 4 days after radiation. These results make sense if the plasma space measured by the $^{125}$I-HSA reflected mainly the blood content of the larger blood vessels. The opening of even a large number of exchange vessels (capillaries) would not contribute greatly to total vascular space, but would considerably increase the surface area available for diffusion and consequently would have a profound effect on $^{86}$Rb uptake.

In a previous communication (Zanelli *et al.*, 1975) it was shown that Nembutal causes a reduction in muscle relative blood perfusion by a factor of about 3·5, and an increase in tumour relative blood perfusion by a factor varying between 1·8 and 3·2 (depending on the type of tumour). The present results (Figs. 2 and 3) show that, in irradiated tumours, Nembutal causes a further increase in tumour relative blood perfusion. It was also found (data not shown) that, concomitant with this further increase in tumour relative perfusion, the relative perfusion of the (unirradiated) muscle in the same animals was further reduced. Skin, however, showed little or no effect: presumably, powerful homoeostatic mechanisms act to maintain skin blood perfusion at an adequate level.

A similar set of experiments was carried out in non-tumour-bearing CBA mice in which the hind legs were irradiated to the same set of radiation doses as before. The mice were injected with the radioactive tracers 4 days later, and the muscle and skin removed for counting. The results are shown in Figs. 4 and 5. Irradi-

Fig. 4.—Effect of Nembutal on the relative blood perfusion in skin and muscle of the hind legs of CBA mice 4 days after various doses of X-rays. The bars indicate s.e.

Fig. 5.—Effect of Nembutal on the plasma space in skin and muscle of the hind legs of CBA mice 4 days after various doses of X-rays. The bars indicate s.e.

ation has no significant effect, except in muscle of non-anaesthetized mice at doses of 2000 rad or more. The usual severe drop in relative perfusion in the muscle of mice given Nembutal, is not modified by the radiation. In irradiated skin, there are no significant changes either with or without Nembutal. This is an entirely different picture from that seen in tumours. The small increase in the $^{86}$Rb uptake of muscle in non-anaesthetized mice after high doses of radiation points to vasodilation in this tissue. But muscle must still be under neurogenic control since, under the influence of Nembutal, when the maintenance of an adequate blood supply to the important viscera is of paramount importance, the radiation-induced vasodilation is entirely suppressed. $^{125}$I-HSA plasma space shows a similar pattern: a small increase in both muscle and skin in unanaesthetized mice and no effect after Nembutal.

As stated above, the experiments described were carried out 4 days after irradiation, to allow recovery from stress caused during the irradiation procedures. However, it is important to look at the time course of the increase in $^{86}$Rb uptake of tumours in anaesthetized mice after irradiation. For this purpose, several groups of CBA mice bearing the NT carcinoma, and WHT mice, bearing the WHT osteosarcoma, had their tumours irradiated to 2000 rad. Two groups of each strain of mice were kept as unirradiated controls. Two groups at a time, 1 given Nembutal and 1 kept unanaesthetized, were sacrificed at various intervals after irradiation.

Fig. 6 shows the pattern of regression and regrowth of these tumours after 2000 rad irradiation. The carcinoma begins to regress very soon after irradiation, but has returned to radiation size in about 15 days. The sarcoma, however, continues to grow for 2–3 days after irradiation, decreases to a shallow minimum and then regrows slowly to return to irradiation size at 20–25 days.

Figs. 7 and 8 show the results of the time course experiments. Four days after irradiation, the Nembutal-plus-irradiation effect on the $^{86}$Rb uptake, seemed to have reached a maximum in both tumours. At later times, the tumours seemed to behave differently: relative perfusion decreased in the NT carcinoma but remained elevated in the sarcoma. Again, little or no effect of the radiation was seen in un-

FIG. 6.—Pattern of regression and regrowth of the CBA " NT " carcinoma and WHT osteosarcoma after 2000 rad of X-rays.

FIG. 7.—Effect of Nembutal on relative blood perfusion in the CBA " NT " carcinoma and WHT osteosarcoma as a function of the time (days) after 2000 rad of X-radiation. The points before Day 0 are for unirradiated controls. The bars indicate s.e.

FIG. 8.—Effect of Nembutal on plasma space in the CBA " NT " carcinoma and WHT osteosarcoma tumours as a function of time after 2000 rad of X-radiation. The points before Day 0 are for unirradiated controls. The bars indicate s.e.

anaesthetized mice. The plasma space results (Fig. 8) are interesting. At 4 days after irradiation the results are similar to those in Fig. 2 at 2000 rad, showing little change with or without anaesthetic. The carcinoma, however, shows an increased plasma space at 15–20 days in anaesthetized mice. The sarcoma, by contrast, shows increased plasma space in unanaesthetized mice at 28 days, with a significant spike at 24 h. Wong, Song and Levitt (1973) have shown an increased rate of protein extravasation in the Walker carcinoma 256 at 18–24 h after 2000 rad. Increased vascular leakage with a tendency to oedema could explain both the significant increase at 24 h after irradiation in the osteosarcoma and the fact that this tumour continues to increase in size for a few days after irradiation. Moreover, oedema would increase the extramural pressure on the larger arterioles and venules, which might become constricted if there is a fall in systemic blood pressure. These factors do not apply to the carcinoma, which begins to shrink very soon after irradiation.

### DISCUSSION

In a previous communication (Zanelli and Fowler, 1974) it was shown that the $^{86}$Rb extraction method is valid as a measure of the fractional cardiac putput perfusing tumours and normal mouse tissues in anaesthetized and unanaesthetized mice. Zanelli *et al.*, (1975) have suggested that the tumour circulation is poorly innervated and behaves like an inert reservoir for blood shunted away from other tissues. How do the present results fit in with the " inert reservoir " model of the tumour circulation? There are 3 possible explanations for the large increase in relative tumour perfusion in anaesthetized mice 4–5 days after irradiation. We shall argue that the third is the best hypothesis.

(1) The tumour vasculature reacts to radiation quickly (relative to that of normal tissues) by active vasodilation. This would increase the size of the "inert reservoir" so that, after Nembutal it would be able to accept more of the blood shunted away from other organs (it should be noted that increased vascular permeability, such as reported by Song *et al.* (1966) and Jolles and Harrison (1966) in irradiated normal tissues, would have little effect on the uptake of $^{86}$Rb because it is rapid in any case). This hypothesis does not hold, because a generalized vasodilation in the tumour would have been detected as an increased $^{86}$Rb uptake in the tumour, even in the absence of Nembutal. If anything, the results suggest that after irradiation the relative tumour blood perfusion in non-anaesthetized mice either decreases marginally or remains unaltered.

(2) Cellular death and disruption in the tumour following irradiation stimulates synthesis, or leads to the accumulation of vasoactive substances such as cathecholamines, histamine or prostaglandins. Song *et al.* (1966) found that Indomethacin could prevent the radiation-induced increase in vascular permeability in the dermis and epidermis of cats. Indomethacin is one of the most powerful inhibitors of prostaglandin synthesis (Williams and Morley, 1973; Vane, 1971) and these hormones, which have been implicated in the *local* control of the circulation, have been shown to be produced locally, following various types of stimuli such as trauma, anoxia and various types of cellular injury (Staszewska-Barczak and Vane, 1975). The objections to this hypothesis are the same as in (1): namely, that vasodilation, however bought about, should have resulted in increased $^{86}$Rb uptake in non-anaesthetized mice. The only way around this is to assume that Nembutal aids the synthesis or local accumulation of vasoactive substances—a most unlikely event. However, in order to check on this rather important point, the following experiment was carried out. Several groups of CBA mice bearing the NT carcinoma had their tumours irradiated to 2000 rad and some groups of mice were kept as unirradiated controls. To some groups of mice, Indomethacin was given (10 mg/kg orally) beginning 1 h before irradiation, and then daily until sacrifice 4 days later. The experiments were then conducted as described above with some groups anaesthetized with Nembutal and some left unanaesthetized. A similar experiment was carried out using the potent $\beta$-adrenergic blocking agent Propranolol (10 mg/kg i.p.). Neither Propranolol nor Indomethacin had any significant effect, either on the Nembutal-induced increase in $^{86}$Rb uptake in irradiated tumours, or in unanaesthetized mice, or unirradiated tumours (Indomethacin did cause a small reduction in the $^{86}$Rb uptake in the irradiated tumours after Nembutal, but the effect was not statistically significant).

(3) The third possibility, and the one which best fits the present results as well as those of other authors, is that the tumour vasculature has been damaged by the radiation in such a way that it has become more sensitive to changes in blood pressure. The shunting away of blood from other tissues under the action of Nembutal would then lead to a greater passive expansion in the "inert reservoir" model of the tumour vasculature. The lack of change, or the small decrease in relative tumour blood perfusion following irradiation in unanaesthetized animals would then imply that *in the absence of stress* the tumour vasculature shows little or no *functional* damage. Only when the vasculature is stressed (when functional demands are made upon it) does the damage become evident. Confirmation or otherwise of this last hypothesis must await more detailed studies making use of more refined physiological techniques such as, for example, the measurement of the critical closing pressure of the vascular bed of tumours before and after irradiation.

One point of major practical importance is the difference in functional response to radiation between the vasculature of different tumours and between that of tumours and normal tissues. In different tumours, the *degree* of damage seems to be different, while the time course seems to be more or less the same. In normal tissues, the time-sequence must be different, since at 4 days no significant changes were found. Further experiments are required to elucidate this "time-dose" difference in functional vascular damage between the vasculature of tumours and that of normal tissues.

The implications of the present results to experimental radiobiology are self-evident. Most, if not all, of the experiments involving transplanted animal tumours in this field of research assume, explicitly or implicitly, a certain status of oxygenation of the tumour cells at any time during a sometimes protracted course of treatment. The present results imply that such assumptions may not be justified. Different types of treatment will stress the vasculature differently, both in kind and degree, and intercomparison of treatment modes in these circumstances may produce misleading results.

In view of the large variety of methods at present used in the treatment of cancer (hyperbaric $O_2$, hypoxic cell sensitizers, cytotoxic drugs or any combination of these), and since all treatments are likely to cause a certain amount of stress to the organism, it is urgent that as detailed a knowledge as possible of the functional response of the vasculature to endogenous and exogenous stimuli should become available.

### REFERENCES

ALGIRE, G. H., LEGALLAIS, F. Y. & ANDERSON, B. F. (1954) Vascular Reactions of Normal and Malignant Tissue *In vivo*. VI. The Role of Hypotension in the Action of Components of Podophyllin on Transplanted Sarcoma. *J. natn. Cancer Inst.*, **14**, 879.

CATALAND, S., COHEN, C. & SAPIRSTEIN, L. A. (1962) Relationship between Size and Perfusion Rate of Transplanted Tumours. *J. natn. Cancer Inst.*, **29**, 389.

CATER, D. B. & SILVER, L. A. (1960) Quantitative Measurements of Oxygen Tension in Normal Tissues and in the Tumours of Patients and after Radiotherapy. *Acta radiol.*, **53**, 233.

CATER, D. B., GRIGSON, C. M. B. & WATKINSON, D. A. (1962) Changes of Oxygen Tension in Tumours Induced by Vasoconstrictor and Vasodilator Drugs. *Acta radiol.*, **58**, 401.

CATER, D. B., ADAIR, H. M. & GROVE, C. A. (1966) Effects of Vasomotor Drugs and " Mediators " of the Inflammatory Reaction upon the Oxygen Tension of Tumours and Tumour Blood Flow. *Br. J. Cancer*, **20**, 504.

CATER, D. B. & TAYLOR, C. R. (1966) Inflammatory Changes in Tumour Blood Vessels after Systemic 5-Hydroxytryptamine, Bradykinin, KalliKrein or Lysolecithin. *Br. J. Cancer*, **20**, 517.

GOLDACRE, R. J. & SYLVEN, B. (1962) On the Access of Blood-borne Dyes to Various Tumour Regions. *Cancer, N.Y.*, **16**, 306.

HEWITT, H. B., BLAKE, E. R. & WALDER, A. S. (1976) Critique of the Evidence for Active Host Defence against Cancer based on Personal Studies of 27 Murine Tumours of Spontaneous Origin. *Br. J. Cancer*, **33**, 241.

HILMAS, D. E. & GILLETTE, E. L. (1975) Microvasculature of C3H/Di Mouse Mammary Tumours after X-irradiation. *Radiat. Res.*, **61**, 128.

JOHNSON, R. J. R., ZANELLI, G. D. & FOWLER, J. F. (1976) Changes in Mouse Blood Pressure, Tumour Blood Flow and Tumour Temperatures following Nembutal and Urethane Anaesthesia. *Radiology*, **118**, 697.

JOLLES, B. & HARRISON, R. G. (1966) Enzymic Processes and Vascular Changes in the Skin Radiation Reaction. *Br. J. Radiol.*, **39**, 12.

KALLMAN, R. F., DENARDO, G. L. & STASCH, M. S. (1972) Blood Flow in Irradiated Mouse Sarcoma as Determined by the Clearance of Xenon 133. *Cancer Res.*, **32**, 483.

KRUUV, J. A., INCH, W. R. & MCCREDIE, J. A. (1966) Effects of Breathing Gases Containing Oxygen and Carbon Dioxide at 1 and 3 Atmospheres Pressure on Blood Flow and Oxygenation of Tumours. *Can. J. Physiol. Pharmacol.*, **45**, 49.

KRUUV, J. A., INCH, W. R. & MCCREDIE, J. A. (1967*a*) Blood Flow and Oxygenation of Tumours in Mice. II. Effect of Vasodilator Drugs. *Cancer, N.Y.*, **20**, 60.

KRUUV, J. A., INCH, W. R. & MCCREDIE, J. A. (1967*b*) Blood Flow and Oxygenation of Tumours in Mice. III. Effects of Breathing Amyl-Nitrite in Oxygen on Radiosensitivity of the C3H Tumour. *Cancer, N.Y.*, **20**, 66.

MILNE, N., HILL, R. P. & BUSH, R. S. (1973) Factors affecting Hypoxic KHT Tumour Cells in Mice Breathing $O_2$, $O_2 + CO_2$, or Hyperbaric Oxygen with or without Anaesthesia. *Radiology*, **106**, 663.

OLIVE, P. L. & INCH, W. R. (1973) Effect of Inhaling Oxygen/Carbon Dioxide Mixtures on Oxygenation and Cure by X-rays of the C3H Ba Mouse Mammary Carcinoma. *Radiology*, **106**, 673.

REINHOLD, H. S. (1971) Improved Microcirculation in Irradiated Tumours. *Eur. J. Cancer*, **7**, 273.

REINHOLD, H. S., JOVANOVIC, D., KEYEUX, A., MAISIN, J. F. & DUNJIC, A. (1974) The Influence of Radiation on Blood Vessels and Circulation. *Current Topics in Radiation Research Quarterly*, **10**, 151.

RUBIN, P. & CASARETT, G. (1966) Microcirculation of Tumours. II. The Supervascularized State of Irradiated Regressing Tumours. *Clin Radiol*, **17**, 346.

SAPIRSTEIN, L. A. (1958) Regional Blood Flow by Fractional Distribution of Indicators. *Am. J. Physiol.*, **193(1)**, 161.

SONG, C. W., ANDERSON, R. S. & TABACHNICK, S. (1966) Early Effects of Beta Irradiation on Dermal Vascular Permeability to Plasma Proteins. *Radiat. Res.*, **27**, 604.

SONG, C. W. & LEVITT, S. H. (1970) Effect of X-irradiation on Vascularity of Normal Tissues and Experimental Tumour. *Radiology*, **94**, 445.

SONG, C. W. & LEVITT, S. H. (1971*a*) Vascular Changes in Walker Carcinoma 256 of Rats following X-irradiation. *Radiology*, **100**, 397.

SONG, C. W. & LEVITT, S. H. (1971*b*) Quantitative Study of Vascularity in Walker Carcinoma 256. *Cancer Res.*, **31**, 587.

SONG, C. W. PAYNE, J. T. & LEVITT, S. H. (1972) Vascularity and Blood Flow in X-irradiated Walker Carcinoma 256 of Rats. *Radiology*, **104**, 693.

SONG, C. W., SUNG, J. H., CLEMENT, J. J. & LEVITT, S. H. (1974) Vascular Changes in Neuroblastoma of Mice Following X-irradiation. *Cancer Res.*, **34**, 2344.

STASZEWSKA-BARCZAK, J. & VANE, J. R. (1975) The Role of Prostaglandins in the Local Control of Circulation. *Clin exp. Pharmacol. Physiol.*, Suppl. 2, 71.

URBACH, F. (1961) The Blood Supply of Tumours. In *Advances in Biology of Skin. Vol. II. Blood Vessels and Circulation*. Eds. W. Montagna and R. A. Ellis. Oxford: Pergamon Press. p. 123.

VANE, S. R. (1971) Inhibition of Prostaglandin Synthesis as a Mechanism of Action for Aspirin-like Drugs. *Nature, Lond.*, **231**, 232.

WILLIAMS, T. J. & MORLEY, J. (1973) Prostaglandins as Potentiators of Increased Vascular Permeability in Inflammation. *Nature, Lond.*, **246**, 215.

WONG, H. H., SONG, C. W. & LEVITT, S. H. (1973) Early Changes in the Functional Vasculature of Walker Carcinoma 256 Following Irradiation. *Radiology*, **108**, 429.

ZANELLI, G. D. & FOWLER, J. F. (1974) The Measurement of Blood Perfusion in Experimental Tumours by Uptake of $^{86}$Rb. *Cancer Res.*, **34**, 1451.

ZANELLI, G. D., LUCAS, P. B. & FOWLER, J. F. (1975) The Effect of Anaesthetics on Blood Perfusion in Transplanted Mouse Tumours. *Br. J. Cancer*, **32**, 380.

ZANELLI, G. D. & LUCAS, P. B. (1976) Effects of Stress on Blood Perfusion and Vascular Space in Transplanted Mouse Tumours. *Br. J. Radiol.*. **49**, 382.